# How does the sex composition of children affect men's higher ideal family size preference relative to women and contraceptive use patterns among couples? A cross-sectional analysis of dyadic couple's data in India

**DOI:** 10.1016/j.ssmph.2021.100835

**Published:** 2021-06-06

**Authors:** Arnab K Dey, Rajib Acharya, Shweta Tomar, Jay G Silverman, Anita Raj

**Affiliations:** a Center on Gender Equity and Health, Department of Medicine, University of California, San Diego School of Medicine, La Jolla, CA, USA; b Population Council, New Delhi, India; c Department of Education Studies, Division of Social Sciences, University of California, San Diego School of Medicine, La Jolla, CA, USA

**Keywords:** Ideal family size, Sex composition, Parity, Modern contraceptive use, Couples, India

## Abstract

**Introduction:**

Son preference, an ongoing concern in India, is a known driver of ideal family size preferences and contraceptive use among couples. These associations can vary substantially with parity and can influence men and women differently. This study assesses the association of sex composition of children by parity and a) men's higher ideal family size preference relative to women and b) use of modern contraceptives by couples.

**Methods:**

We used the Couples Recode dataset from National Family Health Survey (NFHS) 2015-16 and identified couples who had at least one child and had complete responses for variables used in the study (N = 56,731 couples). We developed multivariable linear and logistic regression models to study the association between sex composition of children by parity and our dependent variables.

**Results:**

Our findings indicate that the sex composition of children is associated with men's higher ideal family size preference, relative to women, among couples with four or more children. We also find that couples with less than four children are less likely to use modern contraceptives when they have an equal or a greater number of daughters than sons compared to those who have no daughters. Findings suggest that couples with four or more children are more likely to use modern contraceptives when they have at least one son and one daughter and are less likely to use contraceptives when they have all daughters and no sons, than couples who have no daughters.

**Conclusion:**

This study contributes to existing research on the relationship between sex composition of children with ideal family size preferences and contraceptive use by highlighting meaningful differences between higher and lower parity couples. Findings from the study can be used by family planning programs in India to customize family planning counselling messages by both sex composition and parity.

## Highlights

• Men do not have a higher ideal family size preference, relative to women among couples with less than four children.
• Men's higher ideal family size preferenceobserved only among couples with four or more children.
• Couples with less than four children are less likely to use modern contraceptives when they have more daughters than sons.
• Couples with four or more children are less likely to use contraceptives if they have all daughters and no sons.

## Background

The use of modern contraceptives is known to promote the overall health and well-being of women and children. Modern contraceptive methods reduce unplanned pregnancies, prevent high-risk pregnancies among high parity women and contribute towards the aversion of maternal, newborn and child morbidity and mortality (Ahmed et al., 2012; Cleland et al., 2012). This is particularly relevant for Low- and Middle-Income Countries (LMICs) that bear a disproportionate burden of unmet need for family planning, unwanted pregnancies, unsafe abortions, and maternal and infant mortalities (Ronsmans & Graham, 2006; Wulifan et al., 2016). For example, in India, less than 50% of women use any modern method of contraceptives, with the usage being highly skewed towards female sterilization (IIPS & MoHFW, 2015, p. 16). Overall, about 75% of women who use modern contraceptives report having been sterilized. Further, the uptake of modern contraceptives and the proportion of sterilized users vary substantially with parity and the sex composition of children. The variations are especially pronounced among women with higher parity. For example, among women with four or more children, 51% reported using any modern method of contraception with 84% of those being sterilized. These proportions were similar for women with four or more children who had at least one male child. However, when women of the same parity have no sons, the uptake of modern contraceptive uptake reduces from 51% to 34% and the uptake of female sterilization drops from 84% to 69% (IIPS & MoHFW, 2015, p. 16). Such behaviors around family planning adversely impact the health of mothers and children by increasing the chances of high-risk pregnancies and maternal/neonatal mortality.

These patterns point towards a strong association between the sex composition of children and women's desire to have more children. Prior research has shown fertility-related attitudes to be predictive of their reproductive health behaviors and fertility (Freedman et al., 1975; Hagewen & Morgan, 2005; Lightbourne, 1985). Several studies conducted in the South Asian context have identified strong associations between sex composition of children and fertility desire and other reproductive decisions including contraceptive use (Calhoun et al., 2013; Chaudhuri, 2012; Edmeades et al., 2012; Jayaraman et al., 2009; Kastor & Chatterjee, 2018; Kumar et al., 2021; Stash, 1996; Yadav et al., 2020). However, most of these studies, focused on women's fertility preferences with very few studies considering men's fertility preferences alongside that of women. This is despite strong evidence from other contexts, which shows that men play an important and often overbearing role in a couple's reproductive decision-making (Ampofo, 2001; Bankole & Singh, 1998; Becker, 1996; Blanc, 2001; Dodoo, 1995, 1998; Dodoo & Seal, 1994; Dodoo & Van Landewijk, 1996; Dudgeon & Inhorn, 2004; Ezeh et al., 1996; Tomar et al., 2020). Given that fertility preference plays an important role in contraceptive use and fertility, and that men have a significant role in a couple's fertility decision making, it is critical to study the effect of sex composition of children by parity on the relative fertility preferences of both men and women instead of considering women's fertility preference alone.

This study assesses the effect of number of the children by parity on men's higher ideal family size, relative to women, and couples' modern contraceptive use. A deeper understanding of the interplay between couples' ideal family size preference and contraceptive use related to their child sex composition across parity will help in developing a better understanding of the mechanisms that drive couples' fertility decisions. Such an understanding can inform family planning (FP) programs and strategies to improve sexual and reproductive health outcomes among couples in India.

## Methods

### Data source

This study uses the Couples Recode (CR) dataset from the fourth round of India's National Family Health Survey (NFHS) conducted in 2015–2016. The survey was conducted jointly by the Ministry of Health and Family Welfare (MOHFW) and the International Institute of Population Studies (IIPS). The dataset selected for the study consists of one record for each couple who declared to be married (or living together) to each other. The dataset comprised of completed records only and was created by merging individual datasets of men and women. The study design and sampling strategy adopted for the survey have been described in detail in the national report (IIPS & MoHFW, 2015, p. 16). The dataset included 63,696 cases, of which we excluded 6965 cases, most of which were couples who had no children, and a few men and women who did not provide valid responses about their ideal family size. The remaining 56,731 cases were included for analysis. All the estimates from the univariate, bi-variate and multi-variate models in the paper are based on sample weights. However, only the unweighted sample denominators and numbers are provided in the supporting tables for brevity.

### Measures

As a part of the survey, several questions related to family planning and reproductive health were asked to both men and women. For this study, we included items that captured the current use of contraceptive methods by couples as reported by women, ideal family size reported by men and women, number of current living children and other socio-demographic variables including age and years of education of both men and women, household wealth, religion, and place of residence of the couple.

#### Dependent variables

We used men's higher ideal family size preference, relative to women and the current use of modern contraceptives by the couple as the dependent variables in our study. Men's higher ideal family size preference, relative to women was defined as the difference in ideal family size preferences between men and women. The variable was computed by subtracting the ideal family size reported by women from that reported by men and was treated as a continuous variable in our models. The current use of any modern contraceptive method by the couple was captured from women's reports. This variable was coded as a dichotomous variable with code 1 to indicate current users of condoms, oral contraceptive pills, Intra-Uterine Device (IUD), Sterilization, or other methods such as injections, implants, diaphragm, or foam and jelly. The variable was coded 0 if the couple reportedly used no method or relied on traditional contraceptive methods, including periodic abstinence, withdrawal, and lactational amenorrhea.

#### Independent variables

The primary independent variable was sex composition across parity. To capture this variable, we first coded parity as a categorical variable with four levels to identify couples with: one child, two children, three children, and four or more children. To capture sex composition of children across parity, we created unique levels of sex composition for different levels of parity. For couples with one child, we used a dichotomous variable for sex composition to distinguish between couples who had one son and no daughters (Ref) from those who had one daughter and no son. For couples with two children, we created a categorical variable with three levels to capture sex composition of children. This variable identified couples who had a) two sons and no daughters (Ref), b) one daughter and one son and, c) two daughters and no sons. Similarly, for couples with three children, we created a categorical variable with four levels. This variable identified couples who had a) three sons and no daughters (Ref), b) two sons and one daughter, c) one son and two daughters and, d) three daughters and no sons. Finally, for couples who had four or more children, we captured sex composition of children by creating a categorical variable with six categories. This variable identified couples who had a) four or more sons and no daughters (Ref), b) three or more sons and one daughter, c) two or more sons and two daughters, d) one or more sons and three daughters, e) one or more sons and four or more daughters and, f) four or more daughters and no sons.

#### Covariates

Individual and household-level socio-demographic variables were included as covariates in the statistical models. This included age and years of education completed by men and women, their religion, household wealth and place of residence. Age was used as a continuous variable in the analysis. Years of education was also used as a continuous variable in the study and represented the number of years of education completed by men and women. Religion was coded as a dichotomous variable, categorizing respondents as Muslim or Non-Muslim based on their religion. Household wealth was captured using the Standard of Living Index (SLI) used in the NFHS that is a proxy indicator used to assess the economic status of a household (IIPS & MoHFW, 2015, p. 16). The SLI ranged between 0 and 100 and was used as a continuous variable in the models. Place of residence was categorized as Rural or Urban based on the location of the primary sampling units of the respondents.

### Analysis

#### Descriptive analysis

Descriptive statistics were calculated for all the variables in the study using univariate analysis. We further cross-tabulated the sex composition of children by parity across the type of contraceptive method used to study the distribution of contraceptives used by sex composition.

#### Multivariable regressions models

We developed separate multivariable regression models for each of the dependent variables in the study: men's higher ideal family size preference (Model-1) and, current use of any modern contraceptive method by the couple (Model-2). For each of the dependent variables, we created four multivariable regression model corresponding to the four subgroups of couples based on their parity: one child (Models – 1a and 2a), two children (Modes – 1 b and 2 b), three children (Models – 1c and 2c) and four or more children (Models – 1 d and 2 d).

For Models – 1 (a-d), we developed multivariable linear regression models with men's higher ideal family size as the dependent variable and sex composition of children across parity as the independent variable. We adjusted all these models for men's and women's age, their years of education, religion, household wealth and place of residence.

For Models – 2 (a-d), we developed multivariable logistic regression models where we regressed current use of any modern contraceptive method by the couple on the sex composition of children across parity, adjusting for men's higher ideal family size and all the covariates from Models 1. All analyses were conducted using R (version 4.0.4).

## Results

*Sample Characteristics:* Men's age, in the study sample, ranged from 17 to 54 years (mean = 38.48, Std. Dev. = 8.19) and women's age ranged from 15 to 49 years (mean = 33.51, Std. Dev. = 7.73). More than half of the couples (56.83%) reported using any modern method of contraceptives. About a fifth of the couples had a single child (20.92%), 40.44 percent of the couples had two children, 22.06 percent of couples had three children and the remaining 16.58 percent of the couples had four or more children (Table 1).

**Table 1:** Descriptive statistics of modern contraceptive use, men's higher ideal family size, relative to women, and other key covariates (N = 56,731).

		% or mean (SD)
Dependent Variables		
Any modern contraceptive method	No/Traditional Method	43.17
	Any modern method	56.83
Contraceptive method used	No/Traditional Method	43.17
	Condom	6.58
	Oral Contraceptive Pill	4.94
	Intra Uterine Devices	1.80
	Female Sterilization	42.96
	Other modern methods	0.56
Men's higher ideal family size, relative to women	Mean (SD)	0.08 (1.01)
**Primary Independent Variables**		
Current number of living children	One child	20.92
	Two children	40.44
	Three children	22.06
	Four or more children	16.58
**Other covariates**		
Age of women	Mean (SD)	33.51 (7.73)
Age of men	Mean (SD)	38.48 (8.19)
Years of education – Women	Mean (SD)	6.02 (5.15)
Years of education – Men	Mean (SD)	7.52 (4.95)
Religion	Islam	12.17
	Other religions	87.83
Household Wealth	Mean (SD)	47.64 (19.03)
Place of residence	Rural	65.10
	Urban	34.90

### Sex composition of children and contraceptive use

Across all parity, the proportion of couples who had more sons than daughters was greater than the proportion of couples with more daughters than sons. Cross-tabulation of sex composition of children and contraceptive use indicates that among couples with more daughters than sons, a higher proportion does not use any modern method of contraception (Table 2).

**Table 2:** Sex composition of children by parity across the type of contraceptive method used by the couple (N = 56,731).

		Overall (%)	Type of contraception used			
			No method or traditional methods (%)	Condoms or Oral Contraceptive Pills (%)	Intra Uterine Devices (%)	Female Sterilization (%)
One Child	No Daughters, 1 Son	55.8	53.4	59.0	63.7	65.7
	1 Daughter, No Sons	44.2	46.6	41.0	36.3	34.3
Two Children	No Daughters, 2 Sons	29.5	25.4	25.5	26.0	33.3
	1 Daughter, 1 Son	54.6	53.2	55.4	60.2	55.1
	2 Daughters, No Sons	15.9	21.4	19.1	13.8	11.6
Three Children	No Daughters, 3 Sons	11.4	10.8	8.9	8.1	12.2
	1 Daughter, 2 Sons	43.1	37.6	32.8	28.7	47.8
	2 Daughters, 1 Son	38.0	39.6	47.0	56.7	35.4
	3 Daughters, No Sons	7.5	12.0	11.2	6.6	4.6
Four or more Children	No Daughters, 4+ Sons	3.5	3.9	2.8	3.0	3.4
	1 Daughter, 3+ Sons	15.0	15.1	13.8	12.8	15.0
	2 Daughters, 2+ Sons	31.3	27.4	29.9	33.9	35.1
	3 Daughters, 1+ Sons	29.7	28.1	31.3	26.2	31.1
	4+ Daughters, 1+ Sons	16.0	19.0	16.0	13.9	13.4
	4+ Daughters, No Sons	4.5	6.6	6.3	10.2	2.1

N for one child = 11,357; N for two children = 20,980; N for three children = 13,113; N for four or more children = 11,281.

N for No method or traditional methods = 26,611; N for condoms or oral contraceptive pills = 7097; N for Intra Uterine Devices = 1343; N for Female Sterilization = 21,244.

### Findings from multivariable regression models

**Fig. 1:**
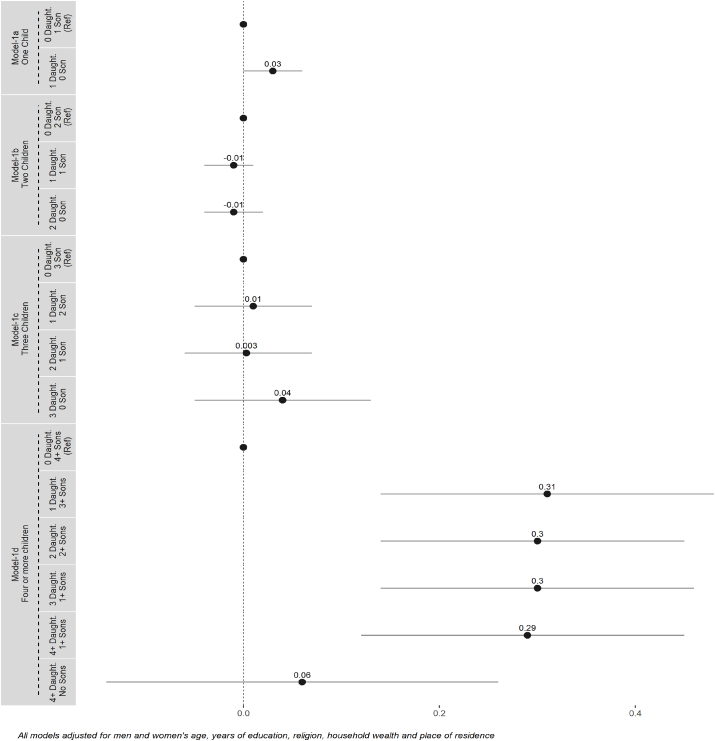
Models 1 (a–d) Coefficients and 95% CIs from multivariable linear regression models with men's higher ideal family size as the dependent variable and sex composition of children by parity as the primary independent variable.

Our multivariable linear regression models 1 (a-d) assess the association between men's higher ideal family size preference and sex composition of children across parity. The models indicate that men's higher ideal family size is not associated with the sex composition of children when couples have less than four children. However, among couples with four or more children, men's higher ideal family size was found to be positively associated with the sex composition of children. Among such couples (with four or more children), men were found to have a higher ideal family size preference relative to women when the couple had: three or more sons and one daughter (β = 0.31, 95% CI: 0.14, 0.48), two or more sons and two daughters (β = 0.30, 95% CI: 0.14, 0.45), one or more sons and three daughters (β = 0.30, 95% CI: 0.14, 0.46) and one or more sons and four or more daughters (β = 0.29, 95% CI: 0.12, 0.45); as compared to those who had four or more sons and no daughters. The association was not significant when the couple had no sons and four or more daughters (models 1 a-d) (Fig. 1, Supplemental Table-B).

Multivariable logistic regression results from models 2 (a-d) capture the association between the current use of modern contraceptive by the couple and sex composition of children across parity.

The models indicate that couples with less than four children are less likely to use modern contraceptives when they have equal or more daughters than sons. Among couples with a single child, those who had a daughter (and no sons) were significantly less likely to use any modern contraceptives as compared to those who had a son (and no daughter) (AOR = 0.75, 95% CI: 0.70, 0.82) (model-3-a). Similarly, among couples with two children, those who had one son and one daughter (AOR = 0.83, 95% CI: 0.78, 0.89) and those who had two daughters and no sons (AOR = 0.50, 95% CI: 0.45, 0.54) were less likely to use contraceptives as compared to those who had two sons and no daughters (model-3-b). Among couples with three children, those who had two daughters and one son (AOR = 0.82, 95% CI: 0.72, 0.94) and those who had three daughters and no sons (AOR = 0.42, 95% CI: 0.36, 0.50) were significantly less likely to use any modern contraceptives as compared to those who had three sons and no daughters (model-3-c).

**Fig. 2:**
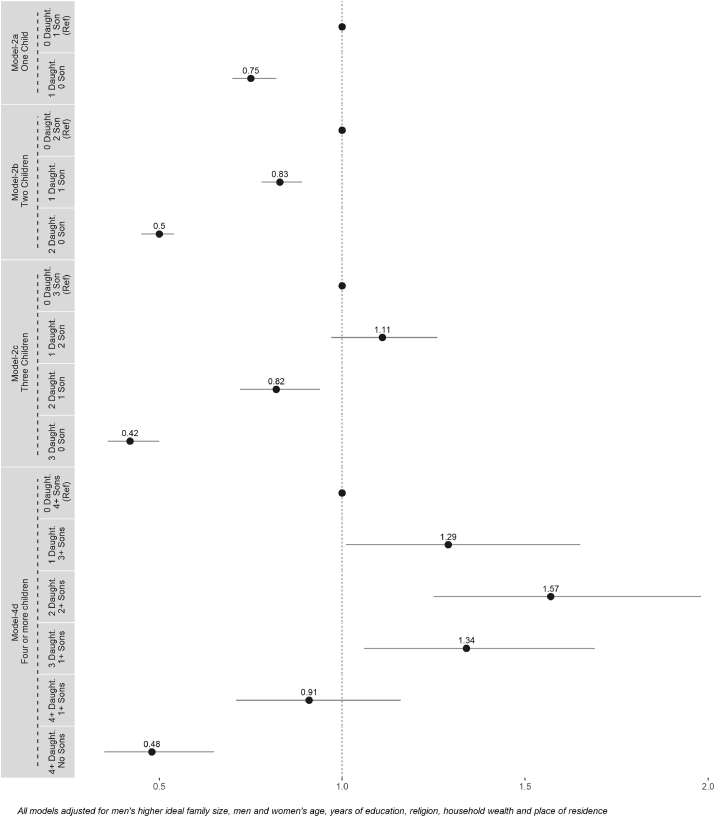
Models 2 (a–d) Adjusted Odds Ratio and 95% CIs from multivariable logistic regression models with current modern contraceptive use as the dependent variable and sex composition of children by parity as the primary independent variable.

In contrast, couples with four or more children were more likely to use modern contraceptives when they had one or more sons and one or more daughters as compared to those who had four or more sons and no daughters. Among these couples (four or more children), those who had three or more sons and one daughter (AOR = 1.29, 95% CI: 1.01, 1.65), those who had two or more sons and two daughters (AOR = 1.57, 95% CI: 1.25, 1.98) and those who had one or more sons and three daughters (AOR = 1.34, 95% CI: 1.06, 1.69) were more likely to use contraceptives as compared to those who had four or more sons and no daughters. However, couples when such couples (four or more children) had four or more daughters and no sons, they were significantly less likely to use modern contraceptives as compared to those who had four or more sons and no daughters (AOR = 0.48, 95% CI: 0.35, 0.65) (models-2 a-d) (Fig. 2; Supplemental Table C).

## Discussion

This study assesses the role of sex composition of children across parity on a) men's higher ideal family size, relative to women and b) modern contraceptive use by couples, with the goal of providing guidance and insight into recommendations for family planning programs in India. Using dyadic data from couples in India, we identify meaningful differences between higher and lower parity couples with regards to the association between sex composition of children and couples' relative ideal family size and contraceptive use.

Among couples with less than four children, we do not find any significant association between sex composition of children and men's higher ideal family size preference, indicating that ideal family size preferences for men and women are closely aligned among low parity couples, irrespective of child sex composition. However, when couples have four or more children, we find that men have a higher ideal family size preference, relative to women for all sex compositions, except those with all daughters and no sons.

We also find that couples with less than four children are less likely to use modern contraceptives when they have equal or more daughters than sons compared to those who have no daughters and all sons. In contrast when couples have four or more children, they are more likely to use contraceptives when they have at least one son and one daughter as compared to those who have no daughters and all sons. Interestingly, couples who have four or more daughters and no sons are significantly less likely to use contraceptives as compared to those who have four or more sons and no daughters.

This work offers interesting insights into the impacts of sex composition by parity on men's higher ideal family size preference and contraceptive use among Indian couples. Prior research focusing on women's fertility desire had shown that women's desire to have another child was positively associated with the number of daughters (Chaudhuri, 2012; Edmeades et al., 2012; Jayaraman et al., 2009; Kumar et al., 2021). Earlier research that captured men's ideal family size preferences has also shown that men are more likely to prefer having more sons at the expense of larger families as compared to women (Dodoo & Seal, 1994; Stash, 1996). Our work now highlights a strong, positive association between sex composition and the husband's higher ideal family size among high parity couples, an increasingly smaller segment of the Indian population. In contrast, the association between sex composition and modern contraceptive use is most notable in couples with less than four children. Our findings, which are consistent with prior research (Chaudhuri, 2012; Edmeades et al., 2012; Jayaraman et al., 2009; Kumar et al., 2021), indicate that among such couples, having more or equal number of daughters as sons is predictive of non-use of modern contraceptives. Our work also adds to the literature by demonstrating that couples with four or more children are more likely to use contraceptives when they have at least one son and less likely to use contraceptives when they have no sons.

These findings suggest that sex composition, specifically having more girls than boys or having no boys, is a key driver of modern contraceptive use in India and early in the process of family creation. However, among high parity couples, vulnerability to husband pressure for more children may be a greater concern for women. Consequently, family planning interventions may need to be tailored to populations by both sex composition and parity, as drivers may differ for these families.

### Strengths and limitations

Studies should be considered in light of their strengths and limitations. Strengths of this study include its use of dyadic data from men and women which allows us to assess the effect of sex composition by parity on men's higher ideal family size, relative to women. The use of dyadic data also allows us to include confounders for both men and women.

The study is not without limitations. As the study uses cross-sectional data, it cannot assume causation. We also could not account for the change in men's ideal family size over time and with changing sex composition of children and could only study it cross-sectionally. Further, the study is also limited as we could not include the perspectives of other family members (e.g., mother-in-law) as confounders in the models, which could influence the association between sex composition and the dependent variables. It also does not include measures of social norms that can predict men's desire for more children. Besides, we were unable to capture a couple's relative strength of sex preference and number preference, which can affect a couple's ideal family size as well as contraceptive use.

Nonetheless, the study makes important contributions to the field by adding to the existing knowledge on the impacts of sex composition of children by parity on men's higher ideal family size and modern contraceptive used by couples. Future research can focus on studying the relative strength of a couple's sex preference and number preference as it relates to their fertility desires and contraceptive decision making and include potential confounders related to social norms and attitudes of other family members explore these associations in more detail.

### Program implications

Family planning programs working with Indian couples can consider the findings from our analysis. Programs can tailor their messages to address the different fertility preferences of lower and higher parity couples. Our findings suggest that the ideal family size preferences are closely aligned between men and women among couples with less than four children. However, such couples are also less likely to use contraceptives when they have an equal or greater number of daughters than sons. For such couples, programs can target both male and female bias towards having more sons. In contrast, among couples with more than four children, men have a significantly higher ideal family size preference as compared to women, which can make women more susceptible to fertility pressures from their husbands. For such couples, programs can consider developing strategies that specifically address fertility preferences for men, especially when couples have all daughters and no sons.

## Conclusion

We believe this work offers an essential contribution to the literature. One would in fact assume that regardless of parity, sex composition would be predictive of men's higher ideal family size relative to women. However, we find this to be true only among higher parity couples. Simultaneously, it is only among higher parity couples that we see sex composition, specifically having at least one son and one daughter, associated with a higher likelihood of modern contraceptive use. In contrast, having all four or more daughters and no sons, is associated with a lower likelihood of contraceptive use. This furthers existing knowledge on the topic by providing a more nuanced understanding of men's ideal family size preference and couples' contraceptive use behavior, as it relates to sex composition of children across parity.

## Ethical considerations

Ethical clearance for the National Family Health Survey 2015-16 was received from IIPS's Ethical Review Board. During the survey, interviewers obtained informed consent from each respondent before the interview.

## Funding

This study was funded by the 10.13039/100000865Bill and Melinda Gates Foundation [Grant Nos. OPP1163682/INV-010695 and INV-002967] and National Institute of Child Health and Human Development [R01HD084453].

## Authorship contributions

AKD undertook the analysis and drafted the manuscript for publication. RA oversaw the analysis and drafting of the manuscript. ST contributed to the analysis and interpretation of findings. JGS contributed to the interpretation of findings and reviewed the manuscript. AR oversaw the analysis, development of the first draft and contributed to reviewing the manuscript for publication.

## Declaration of competing interest

The Authors declare that they have no competing interests.
