# Supplementary material for: How does the sex composition of children affect men's higher ideal family size preference relative to women and contraceptive use patterns among couples? A cross-sectional analysis of dyadic couple's data in India
Source: SSM Popul Health. 2021 Jun 6;15:100835. doi: 10.1016/j.ssmph.2021.100835 (PMC8193613; doi:10.1016/j.ssmph.2021.100835)
Supplement: Multimedia component 1 [file mmc1.docx]

***Supplemental Table-A: Multivariable Logistic Regression model with any modern contraceptive method as the dependent variable, including an interaction term between number of children and number of daughters***

|  | | **Adjusted Odds Ratio** | **95% Lower Confidence Interval** | **95% Upper Confidence Interval** |
| --- | --- | --- | --- | --- |
| Current number of living children | One child | Ref | - | - |
|  | Two children | 2.83 | 2.58 | 3.10 |
|  | Three children | 2.47 | 2.13 | 2.85 |
|  | Four or more children | 1.32 | 1.06 | 1.64 |
| Presence of son | No Sons | Ref | - | - |
|  | At least one son | 1.32 | 1.22 | 1.43 |
| Number of living children * Presence of a son | One child*No Sons | Ref | - | - |
|  | Two children*At least one son | 1.36 | 1.22 | 1.52 |
|  | Three children*At least one son | 1.73 | 1.48 | 2.03 |
|  | Four or more children*At least one son | 1.89 | 1.51 | 2.37 |
| *Model adjusted for husband’s higher ideal family size, men and women’s age, years of education, religion, household wealth and place of residence* | | | | |

***Supplemental Table B: Model 1 (a-d): Multivariable logistic regression to test association between men’s higher ideal family size and sex composition of children***

| **Model** | **Sex Composition** | | **Coefficient** | **95% Lower Confidence Interval** | **95% Upper Confidence Interval** |
| --- | --- | --- | --- | --- | --- |
|  | **Number of Sons** | **Number of Daughters** |  |  |  |
| Model-1a:  1 child  (N = 11357) | 1 | 0 | Ref | - | - |
|  | 0 | 1 | 0.03 | 0.00 | 0.06 |
| Model-1b:  2 children  (N = 20980) | 2 | 0 | Ref | - | - |
|  | 1 | 1 | -0.01 | -0.04 | 0.01 |
|  | 0 | 2 | -0.01 | -0.04 | 0.02 |
| Model-1c:  3 children  (N = 13113) | 3 | 0 | Ref | - | - |
|  | 2 | 1 | 0.01 | -0.05 | 0.07 |
|  | 1 | 2 | 0.003 | -0.06 | 0.07 |
|  | 0 | 3 | 0.04 | -0.05 | 0.13 |
| Model-1d:  4 or more children  (N = 11281) | 4+ | 0 | Ref | - | - |
|  | 3+ | 1 | 0.31 | 0.14 | 0.48 |
|  | 2+ | 2 | 0.30 | 0.14 | 0.45 |
|  | 1+ | 3 | 0.30 | 0.14 | 0.46 |
|  | 1+ | 4+ | 0.29 | 0.12 | 0.45 |
|  | 0 | 4+ | 0.06 | -0.14 | 0.26 |
| *Models 1a–d adjusted for men and women's age, years of education, religion, household wealth and place of residence* | | | | | |

***Supplemental Table C: Model 2 (a-d): Multivariable logistic regression to test association between use of modern contraceptives and sex composition of children***

| **Model** | **Sex Composition** | | **Adjusted Odds Ratio** | **95% Lower Confidence Interval** | **95% Upper Confidence Interval** |
| --- | --- | --- | --- | --- | --- |
|  | **Number of Sons** | **Number of Daughters** |  |  |  |
| Model-3a:  1 child  (N = 11357) | 1 | 0 | Ref | - | - |
|  | 0 | 1 | 0.75 | 0.70 | 0.82 |
| Model-3b:  2 children  (N = 20980) | 2 | 0 | Ref | - | - |
|  | 1 | 1 | 0.83 | 0.78 | 0.89 |
|  | 0 | 2 | 0.50 | 0.45 | 0.54 |
| Model-3c:  3 children  (N = 13113) | 3 | 0 | Ref | - | - |
|  | 2 | 1 | 1.11 | 0.97 | 1.26 |
|  | 1 | 2 | 0.82 | 0.72 | 0.94 |
|  | 0 | 3 | 0.42 | 0.36 | 0.50 |
| Model-3d:  4 or more children  (N = 11281) | 4+ | 0 | Ref | - | - |
|  | 3+ | 1 | 1.29 | 1.01 | 1.65 |
|  | 2+ | 2 | 1.57 | 1.25 | 1.98 |
|  | 1+ | 3 | 1.34 | 1.06 | 1.69 |
|  | 1+ | 4+ | 0.91 | 0.71 | 1.16 |
|  | 0 | 4+ | 0.48 | 0.35 | 0.65 |
| *Models 2 a –d adjusted for men’s higher ideal family size, men and women’s age, years of education, religion, household wealth and place of residence* | | | | | |
